# 

**Published:** 1884-03

**Authors:** 


### The Alabama State Dental Association

Holds its next annual meeting in Bermingham on the 2nd Tuesday of April proximo, at which it is anticipated there will be a large meeting of the profession of the State, and those in the neighboring States are cordially invited to be present. Liberal provision has been made for clinics, to which one day will, probably, be devoted. A number of good operators will be in readiness for this work.

Let all come prepared to make the meeting interesting and profitable.

### Michigan Dental Society.

The twenty-seventh annual meeting of this Society will be held in the city of Detroit, beginning on Wednesday afternoon, March 26th.

This is one of the oldest State Dental Societies in the country. It has accomplished a great work for the profession, not only in the State of Michigan, but for the profession at large. It has, in quite a number of instances, taken the lead in important steps of progress. It has not only done this, but it has ever been on the alert to advocate and sustain any progressive movements irrespective of their origin.

It has two important interests in charge, over which it exercises a fostering care, viz.: The Dental College of the University of Michigan, and the Law regulating the practice of Dentistry in Michigan.

The attention which these require is, by no means, of minor importance; they will each be efficient and successful in proportion as this Society and the profession of Michigan fulfill their responsibility to them. Whatever else is done, or left undone, the welfare of their interests should not be overlooked.

In reference to other matters a good programme has been prepared, and, doubtless, the meeting will be a good one, and it is to be hoped will be largely attended, not only by the members, but by all of the earnest, progressive members of the profession in the State.

### THE DENTAL REGISTER,

A MONTHLY MAGAZINE DEVOTED TO THE INTERESTS OF THE

**DENTAL PROFESSION.**

*Terms Reduced to* $2.00 *Per Year. Single Copies*, 25 *Cents.*

**EDITED BY PROF. J. TAFT, D.D.S.**

AND

**Published by SPENCER & CROCKER, Ohio Dental and Surgical Depot.**

The volume commences in January and closes in December.

The REGISTER being issued monthly is always fresh, therefore Indispensable to the practitioner who desires to keep posted up with the new inventions and improvements which are daily developed. Neither expense nor effort will be spared to give value and variety to its contents Articles will be illustrated with well executed engravings whenever they will add to the interest or assist to explanation.

Its extensive circulation and frequency of issue make it one of the BEST DENTAL ADVERTISING MEDIUMS in the United States.

All subscriptions must begin with January or July.

Local or special notices, five lines or less, will be inserted for $3 each insertion; over five lines, 50 cts. per line.

All advertising bills payable in advance, or at furthest, after the issue of the first number containing the advertisement. All transient advertisements to be paid for in advance.

☞ CONTRIBUTIONS SOLICITED.

Exclusively Editorial Communications should be addressed to the Editor.

The latest number of the REGISTER may be ordered with or without other goods at any time, and all letters should be addressed to

**SPENCER & CROCKER, Ohio Dental & Surgical Depot, Cincinnati, O.**

